# The role of ceramides in the disruption of the cutaneous permeability barrier, a common manifestation of skin disorders

**DOI:** 10.1016/j.jlr.2024.100593

**Published:** 2024-07-11

**Authors:** Kenneth R. Feingold, Peter M. Elias

**Affiliations:** 1 Department of Medicine, University of California San Francisco, San Francisco, CA, USA; 2 Department of Dermatology, Veteran Affairs Health Care System, San Francisco, CA, USA; 3 Department of Dermatology, University of California San Francisco, San Francisco, CA, USA

A cutaneous barrier to the loss of water and electrolytes is required for life on land. This barrier is located in the stratum corneum (SC), the outermost layer of the epidermis, and is mediated by an extracellular hydrophobic lipid matrix with a unique lamellar organization (1, 2). The composition of this lipid matrix by weight is approximately 50% ceramides, 25% cholesterol, and 15%, free fatty acids, with very little phospholipids (a 1:1:1 M ratio of ceramides, cholesterol, and free fatty acids) (1, 2). The ceramides in the SC are unusual and very diverse with a high percentage of very long-chain N-acyl fatty acids (1, 2, 3). Mice deficient in ceramide synthase 3, which has a unique preference for long-chain C26 and C28 acyl-CoAs and plays a key role in the synthesis of ceramides in the outer epidermis, have abnormal lamellar membranes and a defective permeability barrier (1, 3). Moreover, in humans, mutations in ceramide synthase 3 lead to ichthyosis with impaired barrier function (1). These observations demonstrate the importance of long-chain ceramides in the formation of the lamellar membranes that provide the barrier to water and electrolyte movement in the SC.

It should be recognized that other lipids also play a key role in the formation of the lamellar membranes that provide the cutaneous permeability barrier. For example, the essential fatty acid, linoleate, is required for the synthesis of a subgroup of acylceramides (1). In essential fatty acid deficiency, there is an abnormal appearance of the extracellular lipid membranes and perturbed permeability barrier function (1). Topical application of statins that inhibit epidermal cholesterol synthesis or 5-(tetradecyloxy)-2-furancarboxylic acid that inhibits epidermal fatty acid synthesis also perturbs the permeability barrier (1). Additionally, rare mutations involved in both fatty acid and sterol metabolism have been shown to cause ichthyosis with associated abnormal lamellar membranes and impaired permeability barrier function (4). Moreover, as will be discussed below, common skin disorders, such as psoriasis and atopic dermatitis, also demonstrate abnormalities in SC lipids with associated derangements in skin barrier function (2).

Psoriasis is a chronic inflammatory disease that affects approximately 3% of individuals, which is characterized by keratinocyte hyperproliferation and plaques that are erythematous, round-oval, and well-demarcated, of different sizes covered by white-silver scales (5). The pathogenesis of psoriasis has not been fully elucidated but excessive activation of the immune system is thought to be important in the pathogenesis of psoriasis (5). There is a strong genetic influence on the development of psoriasis, and most of the genes associated with psoriasis are related to the immune system (6). The cutaneous permeability barrier is abnormal in lesional skin and in some but not all studies, it is also abnormal in nonlesional skin. Alterations in the ceramide composition (an increase in short-chain and a decrease in long-chain ceramides) and concomitant decreases in skin barrier function are observed in psoriasis and are similar to the abnormalities observed in atopic dermatitis (2, 7). Interestingly, knocking out serine palmitoyltransferase, a critical enzyme for ceramide biosynthesis, resulted in decreased ceramide levels in the epidermis, abnormal barrier function, and a phenotype similar to psoriasis in mice (8).

In the current issue, Rousel *et al.* report the results of a double-blind, randomized controlled trial in which patients with mild-to-severe plaque psoriasis were randomized to guselkumab, an anti-IL23p19 monoclonal antibody, or placebo (9). Permeability barrier function was determined by transepidermal water loss (TEWL) measurements, and untargeted ceramide profiling was performed using liquid chromatography-mass spectrometry after SC was harvested using tape stripping. At baseline, TEWL was increased in lesional skin, but TEWL in nonlesional skin was similar to controls. Guselkumab treatment decreased TEWL levels in lesional skin to control levels but had no effect on nonlesional skin. At baseline, the ceramide profile was similar in controls and nonlesional skin, while the ceramide profile of lesional skin was markedly different. After guselkumab treatment the ceramide profile in lesional skin was similar to skin from healthy controls and nonlesional skin (ie, restored to normal). Guselkumab resulted in an increase in long-chain ceramides and a decrease in short-chain ceramides. In the guselkumab-treated patients, the improvement in TEWL correlated with the normalization of the SC ceramide profile. Additionally, the improvement in the ceramide profile correlated with a decrease in disease severity. This study demonstrates that decreasing inflammation by inhibiting interleukin (IL)-23 activity not only improves disease severity but also normalizes the ceramide profile and restores the permeability barrier.

The precise mechanisms by which cutaneous inflammation alters the ceramide profile of the SC is unknown, but a number of studies have shown that various cytokines that are increased during inflammation have effects on fatty acid elongation and ceramide synthesis (10). Three examples are described below.

• In human keratinocytes, interferon gamma decreased the expression of the elongases of long-chain fatty acids and ceramide synthase 3 resulting in a decrease in the levels of ceramides with long-chain fatty acids (11).
• IL-4/IL-13 inhibit elongase 3 and 6 expression in keratinocyte cultures (12).
• Tumor necrosis factor alpha, IL-1 beta, IL-6, and IL-33 inhibited elongase 3 in keratinocyte cultures (13).

It is therefore likely that inflammation with an increase in proinflammatory cytokines can lead to alterations in gene expression that result in changes in the synthesis of ceramides leading to alterations in the ceramide profile in inflammatory disease states such as psoriasis or atopic dermatitis. Further studies elucidating the role of various cytokines on regulating epidermal ceramide synthesis would be of interest.

Finally, it should be recognized that the permeability barrier disruption that occurs in many skin disorders can stimulate an inflammatory response with an increased production of proinflammatory cytokines. Experimental disruption of the permeability barrier also leads to an increase in the production of proinflammatory cytokines including tumor necrosis factor alpha, IL-1 alpha, and IL-1 beta (14). Thus, skin disorders, such as atopic dermatitis, which are predominantly due to a defective permeability barrier function, will lead to cutaneous inflammation, which will then result in alterations in lipid metabolism leading to changes in the ceramide profile further aggravating permeability barrier function. Conversely, psoriasis, which is likely often due to immunologic dysfunction leading to inflammation, will also result in alterations in lipid metabolism leading to changes in the ceramide profile and defective permeability barrier function that will result in a further increase in inflammation (Fig. 1). Thus, while the initial insult may differ, the pathophysiology may be similar. Finally, topical treatments that restore permeability barrier function will reduce inflammation regardless of the initial inciting event.

**Fig. 1:**
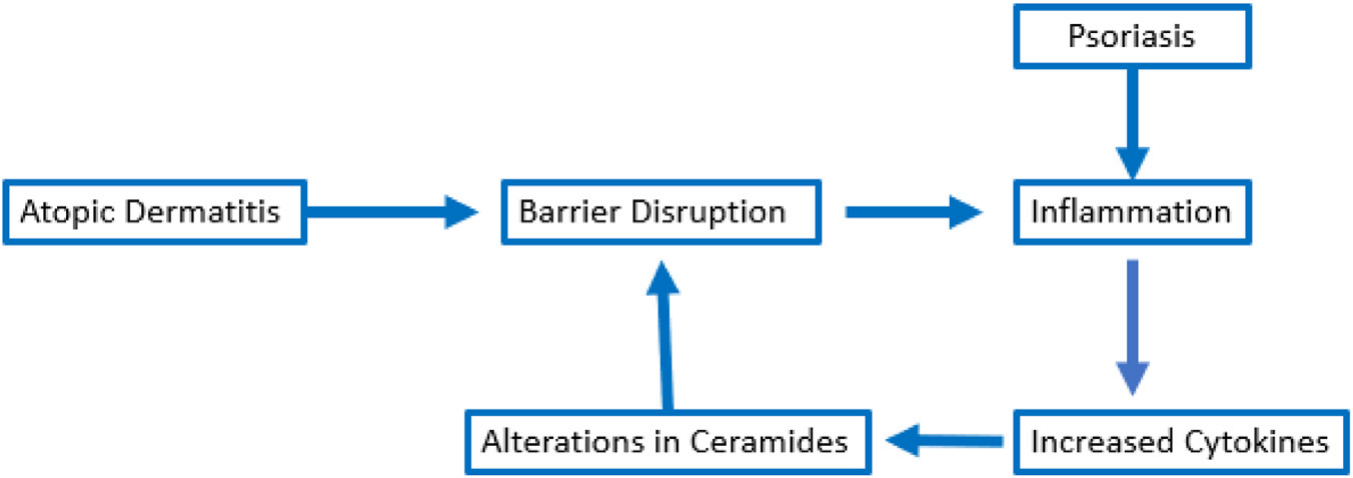
Disruption of the cutaneous permeability barrier disruption in skin disorders. Disruption in the permeability barrier is frequently observed in conjunction with skin disorders. Psoriasis and other inflammatory skin disorders disrupt the barrier by stimulating cytokine production leading to alterations in SC ceramides. Atopic dermatitis and other skin disorders directly alter the permeability barrier, and disruption of the permeability barrier results in inflammation, which will further exacerbate dysfunction of the permeability barrier. Thus, many cutaneous diseases alter permeability barrier function, which then has a positive feedback effect to further worsen permeability barrier function. SC, stratum corneum.

## Conflict of interest

The authors declare that they have no conflicts of interest with the contents of this article.
